# Quantitative approaches in clinical reproductive endocrinology

**DOI:** 10.1016/j.coemr.2022.100421

**Published:** 2022-12

**Authors:** Margaritis Voliotis, Simon Hanassab, Ali Abbara, Thomas Heinis, Waljit S. Dhillo, Krasimira Tsaneva-Atanasova

**Affiliations:** 1 Department of Mathematics and Living Systems Institute, College of Engineering, Mathematics and Physical Sciences, University of Exeter, Exeter, United Kingdom; 2 Section of Endocrinology and Investigative Medicine, Imperial College London, London, United Kingdom; 3 Department of Computing, Imperial College London, London, United Kingdom; 4 UKRI Centre for Doctoral Training in AI for Healthcare, Imperial College London, London, United Kingdom

**Keywords:** Assisted reproductive technology, Machine learning, Mathematical modelling, Pulsatility analysis, Clinical decision making, *In vitro* fertilization, Artificial intelligence, Reproductive endocrinology, Quantitative modelling, HPG, hypothalamic-pituitary gonadal, ART, assisted reproductive technology, GnRH, gonadotropin-releasing hormone, LH, luteinizing hormone, FSH, follicle-stimulating hormone, PCOS, polycystic ovary syndrome, HA, hypothalamic amenorrhea, BSA, Bayesian Spectrum Analysis, AI, artificial intelligence, ML, machine learning, IVF, in vitro fertilization, AMH, anti-Müllerian hormone, OHSS, ovarian hyperstimulation syndrome, E2, estradiol, P4, progesterone

## Abstract

Understanding the human hypothalamic-pituitary-gonadal (HPG) axis presents a major challenge for medical science. Dysregulation of the HPG axis is linked to infertility and a thorough understanding of its dynamic behaviour is necessary to both aid diagnosis and to identify the most appropriate hormonal interventions. Here, we review how quantitative models are being used in the context of clinical reproductive endocrinology to: 1. analyse the secretory patterns of reproductive hormones; 2. evaluate the effect of drugs in fertility treatment; 3. aid in the personalization of assisted reproductive technology (ART). In this review, we demonstrate that quantitative models are indispensable tools enabling us to describe the complex dynamic behaviour of the reproductive axis, refine the treatment of fertility disorders, and predict clinical intervention outcomes.

## Introduction

The reproductive system is a complex endocrine system, involving non-linear feedback and feed-forward interactions conveyed by dynamic hormone signals [1], as well as multifaceted crosstalk with other endocrine axes and the central nervous system [2]. Such complexity makes it challenging to decipher how the system behaves in normal physiological conditions, under acute perturbations, or during chronic disease. To this end, quantitative modelling is an indispensable tool for solidifying our understanding of the system, analysing its dynamic behaviour, and designing medical interventions.

**Figure 1:**
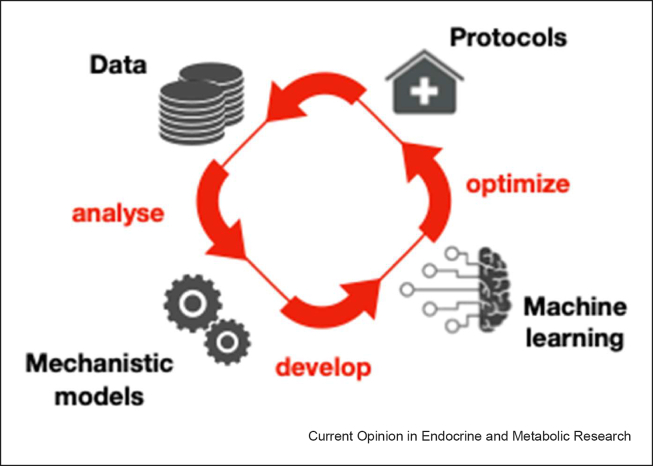
**Utility of quantitative models in reproductive medicine.** This flowchart provides an overview of the workflow of quantitative modelling in reproductive medicine. The first step involves the collection of data, such as hormonal and imaging data. Mathematical models aid the analysis of the data, facilitating extraction of meaningful information. Furthermore, processed data can be used to develop machine learning models with the aim of optimizing current procedures and protocols. The workflow is iterative, enabling continuous model evaluation and improvement.

This review aims to provide an update on how quantitative models are being used in the context of clinical reproductive endocrinology (Figure 1). We focus on computational methods that assist in profiling the dynamics of reproductive hormones, mechanistic models that assist the quantitative assessment of drugs in reproductive medicine, as well as machine learning approaches that are currently used in assisted reproductive technology (ART).

## Computational model for the analysis of hormone pulsatile dynamics

The hypothalamic-pituitary-gonadal (HPG) axis is a complex endocrine system controlling sexual development (throughout fetal, neonatal, and pubertal stages) and reproduction [3]. The system relies on dynamic hormone signals to serve its role. Most notably, gonadotropin-releasing hormone (GnRH) is secreted in a pulsatile manner from the hypothalamus into the anterior pituitary gland, and stimulates the release of gonadotropins (luteinizing hormone, LH; and follicle-stimulating hormone, FSH), which in turn trigger gonadal processes involved in gametogenesis and sex-steroid production [4]. Hence, pulsatile GnRH dynamics are crucial for the onset of puberty and subsequent healthy reproductive function in adults. Disruption in the frequency of GnRH/LH pulses is observed in common reproductive disorders, such as polycystic ovary syndrome (PCOS), in which the frequency and amplitude of GnRH pulses are increased [5], and hypothalamic amenorrhea (HA), in which GnRH pulses are reduced [6]. Therefore, accurate assessment of hormone pulsatility could facilitate the diagnosis and treatment of patients presenting with reproductive endocrine disorders [7].

In clinical research, LH is measured as the gold standard surrogate for GnRH (as it is not possible to measure GnRH in the peripheral circulation at high enough levels). Measuring serum levels of LH at regular intervals (e.g., every 10 minutes) enables quantification and assessment of pulsatile dynamics. However, analysing hormone pulsatility is challenging as pulse-to-pulse variability combined with measurement error often obscure the underlying hormone dynamics [8]. Several computational methods have been proposed in the literature to facilitate the analysis of LH pulsatility [8∗, 9, 10, 11, 12, 13∗∗] (see Table 1). Among these, the deconvolution analysis method is considered the gold standard in clinical research [8]. The method uses a mathematical model describing the time-varying secretion and clearance dynamics of LH, which seeks to fit data and deconvolve the two processes. Data-fitting is achieved via maximum likelihood estimation, providing estimates of the times at which pulses of LH have occurred alongside estimates of the secretion and clearance rates. Bayesian Spectrum Analysis (BSA) presents a different approach to pulsatility analysis, allowing one to quantify the frequency of LH pulses while ignoring mechanistic parameters (e.g., secretion and clearance rates), as well as the actual timing of pulses [14,15]. BSA relies on an abstract model describing generic periodic signals and estimates the frequency from LH data using Bayesian inference [11]. A key strength of the BSA method is that frequency estimates come in the form of Bayesian posterior distributions, facilitating the estimation of uncertainty and hypothesis testing. Finally, Bayesian extensions to the deconvolution method [13,16, 17, 18] as well as a recently proposed Bayesian framework for inference of LH dynamics based on mechanistic models of pulse generation [19] enable uncertainty estimation of parameters as well as estimation of latent hypothalamic dynamics.

**Table 1:** Summary of methods used in LH pulsatility analysis.

Method/Tool	Model	Outputs	Open-source Implementation	Reference
Deconvolution analysis	Mechanistic model	Position of pulses and pulse parameters (point estimates)	*Unavailable*	[8]
Cluster analysis	Statistical pattern matching	Position of pulses (point estimates)	*Unavailable*	[9]
DynPeak	Mechanistic model	Position of pulses (point estimates)	Python	[10]
BaSAR	Harmonic functions	Pulse frequency (posterior distribution)	R package	[11]
Bayesian Deconvolution Analysis	Mechanistic model	Position of pulses; pulse parameters (posterior distribution)	*Unavailable*	[13]
HormoneBayes	Mechanistic model	Model parameters; position of pulses (posterior distribution)	C++	[19]

### The potential of artificial intelligence in assisted reproductive technology

The broad field of artificial intelligence (AI) encompasses machine learning (ML), which specifically refers to statistical models that are leveraged to automatically detect patterns from large and complex datasets in order to make predictions regarding an outcome of interest [20]. AI and ML methods have a wide scope for improving ART [21, 22∗, 23, 24], which includes *in vitro* fertilization (IVF) treatment; a procedure that, for example, inherently requires the classification and selection of both male and female gametes, as well as several complex decisions that are made during the cycle with respect to the dosage, and timing, of hormonal interventions.

Key for the successful application of ML is high-quality substantial datasets that contain strong predictors, capture the variance in the population, and are accurately annotated [25,26]. For this reason, early ML models of predicting live birth after IVF treatment using neural networks achieved a modest accuracy (59%) [27], as they relied on small datasets lacking key predictors. More recently, the accuracy of predictive models trained on richer datasets has increased to 84.4% [28]. Even where ML techniques provide an ability to predict outcomes, some methodologies can remain unexplainable (‘black-box’) [26], such that mechanistic insights into the decision processes carried out by such models may not be evident. Others harness more interpretable methods e.g., random forests [29,30] or linear regression [31], where the most important predictors can be identified. For example, top predictors of live birth after IVF treatment included female partner age, anti-Müllerian hormone (AMH) [32], number of high-quality embryos, and serum estradiol level (reflective of cumulative follicle size and, in turn, the number of eggs that will be retrieved) on the day of administration of the trigger for oocyte maturation [33].

With the recent influx of literature surrounding the use of AI and ML in ART, there is a clear interest in the academic community on how such models can be used to improve treatment strategies in clinical workflows [34].

## AI to support decision-making in *in* *vitro* fertilization

*In vitro* fertilization (IVF) is a complex procedure involving hormonal interventions to act upon specific processes during the treatment cycle. These include: 1. Ovarian stimulation [35], 2. Prevention of premature ovulation [36], 3. Induction of oocyte maturation [29,30], 4. Fertilization *in vitro* and embryo selection for transfer [21,23,24], to hopefully result in live birth [37]. The timings of these interventions can vary depending on the specific IVF protocol carried out by the clinician [38]. In the initial stages of IVF, preparations containing FSH are used to induce the growth of multiple ovarian follicles, whilst a GnRH antagonist, or continuous non-pulsatile administration of a GnRH agonist (which desensitizes the GnRH receptor), is used to prevent a premature LH surge and, in turn, untimely ovulation [38]. Once the follicles grow to the required size, a hormonal trigger, namely either human chorionic gonadotropin (hCG) or a GnRH agonist, is administered to provide LH-like exposure and induce oocyte maturation (i.e., oocytes attain the capacity for fertilization by losing half of their genetic material as the polar body) [38].

**Figure 2:**
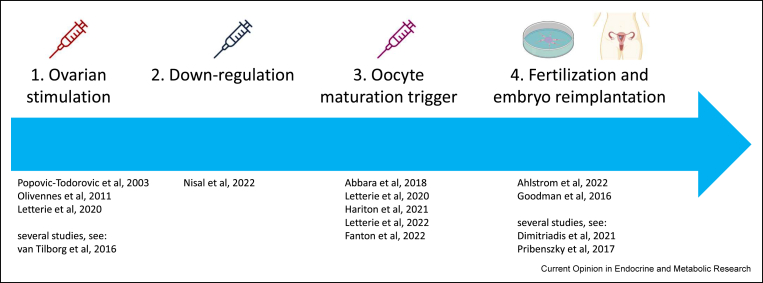
**Potential AI-based interventions during IVFtreatment.** This pipeline outlines the processes carried out during IVF treatment cycles, where interventions using AI and ML techniques could be used to support clinical decision-making. The references provided at each stage indicate literature exploring efforts in quantitative modelling of these stages. The four stages in the figure above correspond to the numbered sections under ‘AI to support decision-making in in vitro fertilization’. Of the four stages presented, the first three pertain endocrinological interventions, where optimizations with respect to dose and timing are of value.

The vast amount of complex data generated before and during an IVF treatment cycle has the potential to be analysed more precisely and objectively using ML techniques. Consequently, there are several processes in the IVF cycle wherein decision-making can potentially benefit from AI pipelines (Figure 2), and have been explored in recent literature [39,40].

### Selection of gonadotropin doses for ovarian stimulation

Quantitative modelling can aid in the selection of the appropriate dose of gonadotropins for ovarian stimulation as the ovarian response to the same dose can vary by baseline characteristics such as age and ovarian reserve (represented by AMH level [32] or antral follicle count [41]). There are several algorithms derived to estimate the optimal initial dose of FSH for ovarian stimulation taking into account baseline factors [42,43]. Studies using such algorithms, and other markers reflective of ovarian reserve [44, 45, 46, 47, 48], have been explored in a systematic review by van Tilborg et al. [49]. Excessive dosing can increase the risk of ovarian hyperstimulation syndrome (OHSS), whereas insufficient dosing can increase the risk of a suboptimal ovarian response [50]. Furthermore, a physician's reaction to an insufficient initial response with a subsequent increase in dose can increase variability in follicle sizes and hamper response to triggering oocyte maturation [35]. Therefore, using AI to optimize the initial dose, and subsequent dose adjustment [40], is likely to improve the success of treatment, although the extent of its impact on later outcomes (e.g., live birth rate) remains undetermined [50].

### Prevention of premature ovulation

Accurate measurement of LH, FSH, estradiol (E2), and progesterone (P4) levels across the normal cycle facilitated the development of a mechanistic mathematical model of the human menstrual cycle [51], incorporating key interactions in the HPG axis. This model described how timing and dosing of GnRH analogues affect hormonal responses: reproducing clinical findings of Nafarelin (GnRH agonist) delaying ovulation when administered in the early follicular phase, while immediately triggering ovulation if administered in the late follicular phase [52]; and predicting that the length of the delay in ovulation after Cetrorelix (GnRH antagonist) administration in the follicular phase depends on the dose used [53].

Nagaraja et al. modelled the inhibitory effect of Cetrorelix (GnRH antagonist) on LH secretion, as well as the induced delay of the LH surge based on the pharmacokinetics of the drug [36,54,55]. Later mathematical models also incorporated mechanistic features of the HPG axis (such as feedback control from the gonads), hence providing a more complete description of the endocrine system and predicting the response to both GnRH agonists and antagonists [56].

Further, in the context of specifically using a GnRH antagonist for pituitary downregulation during IVF treatment cycles, Nisal et al. were able to present the potential application of a quantitative FSH dosing algorithm in a local pilot study [57]. There is scope for the dose and timing of GnRH antagonist to be personalized according to patient characteristics, using more sophisticated AI and ML techniques. Optimized approaches to dose and timing of downregulatory protocols have the potential to reduce costs whilst maintaining, or even improving, pregnancy outcomes as both over and under-suppression of endogenous LH levels can be deleterious.

### Induction of oocyte maturation

The trigger to induce oocyte maturation is administered once follicles grow to the required size to be able to respond appropriately and yield oocytes. Typically, simple rules are used to guide the timing of this step, such as the presence of at least two to three follicles more than 17 or 18 mm in diameter. However, this approach assumes uniform growth of the follicles behind these lead follicles, rather than a more diverse set of follicle sizes [35]. By harnessing ML techniques such as bagged decision trees [58], random forests [30], and linear regression [31], found in the literature, the size of follicles on the day of trigger most likely to yield oocytes has been estimated, and indicates the potential to support the optimization of the timing of trigger administration during clinical workflows [39]. Identification of this follicle size range enables the quantification of oocyte maturation [29], and can provide a target for response to gonadotropins when evaluating response to ovarian stimulation. In essence, ML techniques have the potential to increase the precision, objectivity, and reproducibility of decision-making during IVF protocols.

### Embryo selection for transfer

An example of complex data generated during IVF treatment is image analysis of embryos growing over several days assessed via time-lapse technology, which has the potential to aid in the selection of embryos that are most likely to implant. This represents a large amount of data which can be challenging and impractical for an embryologist to capture manually [21,22]. Additionally, prediction of outcomes based on oocyte quality has been attempted based on their morphology [59,60], texture [61, 62, 63], and morpho-kinetic [64] information. Furthermore, researchers have shown that the mechanical properties of human zygotes are predictive of embryo survival during the blastocyst stage, allowing one to predict within hours after fertilization whether the zygote will arrest with 90% precision [65]. However, the benefit of using AI technology in the embryo selection process has yet to be proven as superior to current means in double-blind randomized controlled trials [66,67], whereby no significant improvement was shown in clinical pregnancy rates when selecting day five blastocysts for transfer with a time-lapse algorithm. These studies highlight the necessity for the accuracy of predictions made via ML techniques to be prospectively tested and validated prior to adoption into clinical practice, with appropriate mitigations of study biases [68].

## Conclusions

Quantitative models enable data-driven support in clinical decision-making. In the context of reproductive endocrinology, mechanistic mathematical models enable the analysis of hormone data and the effect of endocrine interventions, while ML models can facilitate outcome predictions in ART protocols.

Importantly, quantitative models enable us to move away from one-size-fits-all approaches and design patient-optimized protocols. Ultimately, this can reduce operational costs by improving the efficiency and efficacy of treatment to further enhance treatment outcomes, and reduce psychological morbidity associated with unsuccessful treatment. The use of AI in this context remains nascent, however, is expected to continue to burgeon with the inclusion of large diverse multi-center datasets to ensure model generalizability, undergo appropriate validation studies, as well as presenting viable integration into well-established clinical workflows [26].

## Editorial disclosure

Given their role as Guest Editor, Krasimira Tsaneva-Atanasova and Margaritis Voliotis had no involvement in the peer review of the article and has no access to information regarding its peer-review. Full responsibility for the editorial process of this article was delegated to Karen Chapman.

## Conflict of interest statement

Nothing declared.

## Data Availability

No data was used for the research described in the article.
